# A prospective study of smoking-related white blood cell DNA methylation markers and risk of bladder cancer

**DOI:** 10.1007/s10654-024-01110-y

**Published:** 2024-03-30

**Authors:** Roel Vermeulen, Barbara Bodinier, Sonia Dagnino, Rin Wada, Xuting Wang, Debra Silverman, Demetrius Albanes, Neal Freedman, Mohammad Rahman, Douglas Bell, Marc Chadeau-Hyam, Nathaniel Rothman

**Affiliations:** 1 Institute for Risk Assessment Sciences, Division of Environmental Epidemiology, Utrecht University, PO Box 80178, 3508 TD Utrecht, The Netherlands; 2 Faculty of Medicine, School of Public Health, Department of Epidemiology and Biostatistics, Imperial College London, London, UK; 3 MRC Centre for Environment and Health, Imperial College London, London, UK; 4 Commissariat À L’Energie Atomique Et Aux Énergies Alternatives (CEA), Institut Des Sciences du Vivant Fréderic Joliot, Université Côte d’Azur, Nice, France; 5 Immunity Inflammation and Disease Laboratory, National Institute of Environmental Health Sciences, National Institutes of Health, RTP, Durham, NC USA; 6 Division of Cancer Epidemiology and Genetics, National Cancer Institute, National Institutes of Health, Rockville, MD USA; 7 Division of Cancer Control and Population Sciences, National Cancer Institute, National Institutes of Health, Rockville, MD USA

**Keywords:** Bladder cancer, Tobacco use, DNA methylation, Prospective study

## Abstract

Bladder cancer, a common neoplasm, is primarily caused by tobacco smoking. Epigenetic alterations including DNA methylation have the potential to be used as prospective markers of increased risk, particularly in at-risk populations such as smokers. We aimed to investigate the potential of smoking-related white blood cell (WBC) methylation markers to contribute to an increase in bladder cancer risk prediction over classical questionnaire-based smoking metrics (i.e., duration, intensity, packyears) in a nested case–control study within the prospective prostate, lung, colorectal, and ovarian (PLCO) Cancer Screening Trial and the alpha-tocopherol, beta-carotene cancer (ATBC) Prevention Study (789 cases; 849 controls). We identified 200 differentially methylated sites associated with smoking status and 28 significantly associated (after correction for multiple testing) with bladder cancer risk among 2670 previously reported smoking-related cytosine–phosphate–guanines sites (CpGs). Similar patterns were observed across cohorts. Receiver operating characteristic (ROC) analyses indicated that cg05575921 (AHHR), the strongest smoking-related association we identified for bladder cancer risk, alone yielded similar predictive performance (AUC: 0.60) than classical smoking metrics (AUC: 0.59–0.62). Best prediction was achieved by including the first principal component (PC1) from the 200 smoking-related CpGs alongside smoking metrics (AUC: 0.63–0.65). Further, PC1 remained significantly associated with elevated bladder cancer risk after adjusting for smoking metrics. These findings suggest DNA methylation profiles reflect aspects of tobacco smoke exposure in addition to those captured by smoking duration, intensity and packyears, and/or individual susceptibility relevant to bladder cancer etiology, warranting further investigation.

**Supplementary Information:**

The online version contains supplementary material available at 10.1007/s10654-024-01110-y.

## Introduction

Tobacco smoking is the most important risk factor for bladder cancer causing the majority of bladder cancers in both men and women [1]. Smokers are at least 3 times as likely to get bladder cancer as non-smokers [2]. This association is thought to be more driven by smoking duration than intensity of smoking with individuals that smoke a long duration at a low intensity having a greater risk than individuals smoking for a short duration at a high intensity within equal pack-year categories [3]. Although smoking is a well-established risk factor for bladder cancer, current prediction algorithms perform only modestly and prediction improvement could be yielded by including additional risk factors and potential effect modifiers [4–6].

It is well accepted that a history of smoking results in an epigenetic signature of altered DNA methylation at multiple cytosine–phosphate–guanine sites (CpGs). The identification of these smoking-related CpGs allows subsequent investigation of their association with smoking-related morbidities either as independent smoking proxies or as mediators [7–12]. Yu et al. (2021) explored if a risk score based on smoking-related CpGs could increase prediction of bladder cancer as compared to using classical smoking metrics (i.e., duration, intensity, packyears) [6]. Although blood-based DNA methylation markers for smoking were found to be associated with risk of bladder cancer independently of self-reported smoking history, the increase in disease prediction performance was negligible.

We report here on a nested case–control study of bladder cancer within two prospective cohorts. First, we explored the association between the previously reported methylation signature of smoking and compared these results with conventional metrics of smoking (i.e., duration, intensity, packyears). Subsequently, we performed an analysis of DNA methylation of all CpG sites to explore if there was evidence of CpGs or differential methylated regions (DMRs) that associate with bladder cancer risk that are not directly related to smoking.

## Methods

### Study population

Our study population consisted of two nested case–control studies, as described previously [13]: the prostate, lung, colorectal, and ovarian cancer (PLCO) screening Trial, a multi-centre intervention trial with recruitment between 1993 and 2001 including men and women aged 55–74 years old [14, 15]; and the Alpha-Tocopherol, Beta-Carotene (ATBC) Cancer Prevention Study [16], a randomised placebo-controlled trial which took place in Finland between 1985 to 1988 and included men aged 50–69 years old who were active smokers at enrollment. In PLCO incident bladder cancer cases (*n* = 307) were identified using the International Classification of Diseases for Oncology [ICD-O-3] codes C67.0-C67.9. Incident bladder cancer cases (*n* = 482) selected from the ATBC study were defined as histologically confirmed primary carcinoma of the urinary bladder (ICD9 codes 188.1–188.9). In both studies, healthy controls (*N* = 315 for PLCO and *N* = 534 for ATBC) were frequency-matched to cases on sex and 5-year age categories. All PLCO and ATBC participants completed a baseline questionnaire, which included information on age, sex, and smoking. The self-reported smoking information included: (i) the smoking intensity (number of cigarettes smoked per day), and (ii) the smoking duration (in years).

Biospecimen collection for PLCO participants was approved by the US National Cancer Institute (NCI) Special Studies Institutional Review Board (IRB) (OH-C-N041), the US National Institutes of Health, and the IRB at each screening site. Written informed consent was obtained from each ATBC participant, and the study was approved by the IRB of the US NCI and the National Public Health Institute of Finland. The trial was registered as Clinical Trials.gov number NCT00342992 (ClinicalTrials.gov).

### DNA methylation data

DNA methylation data was obtained using the Infinium HumanMethylation450 BeadChip assay. As detailed elsewhere [17], genomic DNA was extracted from pre-diagnostically collected blood samples and underwent hybridisation on HM450 BeadChips according to the manufacturer’s protocol after bisulphite conversion. Hybridised micro-arrays were scanned using Illumina HiScanSQ system, and raw intensity data were exported to Illumina GenomeStudio (version 2011.1). Using control probes from the micro-array, we assessed the efficiency of the bisulphite conversion and excluded those with detection *p*-values greater than 0.05. Further data pre-processing was performed using in-house software for the R statistical computing environment. We censored DNA methylation measurements if obtained by averaging intensities over less than three beads, or if averaged intensities were below detection thresholds estimated from negative control probes. Background subtraction and dye bias correction were also performed. DNA-methylation level at each CpG locus was measured as M-values (logit_2_-transformation of $\beta$-values, the per-site methylation fraction). We excluded (i) samples with methylation values missing for more than 30% of the assayed CpG sites, and (ii) CpG sites in which more than 30% of the measurements were missing. Methylation data from participants of both cohorts were generated in the same lab and pre-processed following the same unified protocol. This standardized approach has been used in previous analyses combining data from multiple studies and ensured comparability of methylation data across studies [18]. Variability due to batch/technical confounding was accounted for using linear mixed models as detailed below.

### Statistical analyses

To maximise the sample size and increase the exposure contrast all statistical analyses, unless otherwise stated, were performed on the pooled dataset including participants from both studies.

### Univariate association study

As proposed previously [17], we sought to identify differentially methylated CpG sites in bladder cancer cases and controls using the following linear mixed model:

1 $$ Y^{i} = \alpha + \beta_{1} X^{i} + \beta_{2} FE^{i} + u^{{ID^{i} }} + u^{{T^{i} }} + \varepsilon^{i} {,} $$

where ${Y}^{i}$ is the measured methylation M value at a given CpG site. ${X}^{i}$ is the (prospective) case control status for individual $i$, and ${\beta }_{1}$ represents the regression coefficient measuring the effect of case–control status on methylation levels. ${FE}^{i}$ is a vector of fixed effect observations for individual $i$, including age at blood collection, gender, and recruitment center, and ${\beta }_{2}$ represents a vector of regression coefficients measuring the effect of each of these factors on methylation levels. Nuisance variation due to technical differences while processing the biosamples was modelled by introducing a random intercept for the chip ID (159 modalities) (${u}^{{ID}^{i}}$), and for the position of the sample on the chip (12 modalities) (${u}^{{T}^{i}}$) [8, 19–21]. For numerical stability, statistical significance of the effect linking the case–control status (${\beta }_{1})$ for each CpG site was inferred using a likelihood ratio test comparing models with and without the variable of interest [19, 22, 23]. As a sensitivity analysis, we ran the same model adjusting for blood cell composition, estimated from a reference-free method.

### Investigating the effect of smoking on bladder cancer

Using an unconditional logistic model, we evaluated the association between smoking metrics and risk of bladder cancer. These included smoking duration (in years), lifelong cumulative smoking exposure (in pack-years), smoking intensity (in packs/day). These were recoded into quartiles based on the control population to ensure that the reference distribution of the exposure to tobacco smoke was representative of that in the overall cohort. To evaluate if the effect of the smoking metrics on the risk of bladder cancer could be explained fully by the smoking-related CpGs we fitted, for each of the three smoking metrics (duration, intensity, or pack-years), a logistic model including the smoking metric alone, and subsequently adjusted for (i) methylation levels at the CpG site showing the strongest association with smoking in our data and (ii) the scores of the first principal component of all CpG sites found associated with smoking status in our data. Conversely, to assess if smoking-related CpG sites were reflecting disease-relevant information that was not captured by the classical smoking metrics, we fitted a logistic model with the (i) methylation levels at the CpG site showing the strongest association with smoking in our data and (ii) the scores of the first principal component of the CpG sites found associated with smoking status in our data as predictors. These models were subsequently adjusted for each of the smoking metrics (duration, intensity, or pack-years). Model performances were evaluated through the area under the curve (AUC) of the received operating characteric (ROC) curve, which was derived using a sub-sampling procedure, where 80% of the population was used to train the model and the remaining 20% to test it. We report the AUC from the testing set for each model investigated.

### Investigating previously identified smoking-related CpGs and bladder cancer

We first restricted our univariate analyses to the CpG sites that were recently reported to be related to smoking status (current or former versus never smokers) [9] and were assayed in our samples after probe filtering. These included a list of 2670 unique CpG sites of which 2,623 CpG sites were found differentially methylated in never vs. current smokers, and 273 in never vs. former comparisons. [9]

To facilitate results interpretation, the methylation levels (M-values) at each smoking-related CpG site found differentially methylated in cases and controls were recoded into quartiles, and odds ratios were calculated (setting the lowest quartile as reference). We also adjusted on the most significant CpG associated with smoking in our dataset and on the first principal component (PC1) summarising the methylation levels at the 2670 smoking-related CpG sites.

Of the (*N* = 2670) smoking-related CpG sites, we investigated those that were differentially methylated in our data (at a Bonferroni corrected significance level ensuring a family-wise error rate of 0.05) and summarised them using a principal component analysis (PCA) of their methylation M values. Components derived from this analysis were subsequently used as a proxy for our data's methylation response to smoking. They were either included in models as confounding factors or related to bladder cancer risk in logistic models.

To account for nuisance variation in the methylation data [21, 22, 24], we ran a linear mixed model setting technical confounders as random intercepts. We subtracted the estimated random intercepts from the observed methylation levels. We used these methylation data in subsequent analyses.

### Epigenome-wide analyses of bladder cancer

In a second stage, univariate analyses were extended from the smoking-related CpG sites to all assayed CpG sites. We corrected both analyses for multiple testing using Bonferroni-corrected per test significance level ${\alpha }^{\prime}$ ensuring a FWER below 0.05 (${\alpha }^{\prime}=\frac{0.05}{{N}_{1}}\text{, and} \frac{0.05}{{N}_{2}}=1.09\times {10}^{-7}\text{, where} {N}_{1}{\text{and}} {N}_{2}$ are the number of the smoking-related and total number of CpG sites assayed in our study population, respectively). As a series of sensitivity analyses, we further adjusted our models on the three different smoking exposure measures. We also stratified our analyses by study, running the model on PLCO and ATBC participants separately, and ran our model restricting the study population to current smokers only.

### Investigating differentially methylated regions (DMRs)

As a multivariate alternative to our univariate screening, we investigated potential DMRs in relation to bladder cancer status using Gaussian kernel smoothing of the T-statistic measuring the per-CpG changes in methylation levels between two sup-populations (here prospective cases and controls) [25]. As implemented in the DMRcate R-package, limma was used to compute T-statistics measuring the association between each individual CpG sites and the variable of interest (here case–control status). Gaussian smoothing weighting of these statistics was performed to evaluate the per CpG site statistical significance, correcting for multiple testing. Per-site significance level was subsequently agglomerated to identify contiguous genomic regions (of variable size) that were enriched in outcome-relevant CpG sites. To account for nuisance variation we used the same ‘de-noised’ data, as described above. To preserve sample size, missing methylation levels were imputed using the K nearest neighbours approach (setting k = 10) [26]. Assuming that the strength of association between two CpG sites is directly related to their functional proximity, we evaluated the relationship between the CpG sites involved in the DMRs and smoking by adopting a network approach. Our network approach was based on the pairwise correlation coefficients linking all CpG sites involved in the identified DMRs and smoking metrics. To induce sparsity in the graph, we only considered correlations (edges) with a Fisher *z*-test *p*-value below the Bonferroni corrected threshold $\left(p<\frac{0.05}{{N}_{test}}];where {N}_{test}=\frac{{N}_{2}\times \left({N}_{2}+1\right)}{2}\right)$. We assumed that the ‘distance’ to smoking, as measured by the length (i.e., steps) of the shortest path linking CpG sites in a DMR and smoking reflected their functional proximity to the exposure. This resulted in the following classification of the CpG sites contributing to the identified DMRs:

- The smoking-related CpG sites that are within the 2670 established smoking-related CpG sites,
- The order 1 CpG sites correlated to at least one smoking-related CpG but not directly to smoking,
- The order 2 CpG sites are correlated to at least one order 1 CpG but not directly to any smoking-related site and not to smoking.

## Results

### Study population and methylation data

Of the 1638 participants included initially in our study, 40 had more than 30% of the methylation data missing (14 from ATBC, and 26 from PLCO) and were excluded from the analyses, leaving us with a population size of 1598 participants. These included 766 bladder cancer cases (288 from PLCO and 478 from ATBC) and 832 controls (308 and 524 in PLCO and ATBC, respectively). Their characteristics are summarised in Table 1 and show that participants were between 49 and 74 years old at recruitment. All 1002 ATBC participants were males and current smokers at recruitment. In the full population, more than 90% of participants were males, and more than 65% were current smokers. As expected, none of the frequency-matching criteria (age at recruitement, and gender) differed between cases and controls and differences could only be observed for smoking exposure variables. After filtering CpG sites due to missing values, 25,393 were excluded (including one smoking-related CpG site) leaving 2670 smoking-related CpG sites and 460,119 CpG sites for our analyses.

**Table 1:** Study population description by gender, age, age at recruitment and smoking exposure variables

Study	Full sample					PLCO					ATBC				
Characteristics	Cases		Controls		*P*^a^	Cases		Controls		*P*^a^	Cases		Controls		*P*^a^
	*N*	(%)	*N*	(%)		*N*	(%)	*N*	(%)		*N*	(%)	*N*	(%)	
*Total*	766	47.9	832	52.1		288	48.3	308	51.7		478	47.7	524	52.3	
*Sex*					0.568					0.604					
Male	704	91.9	771	92.7		226	78.5	247	80.2		478	100	524	100	
Female	62	8.1	61	7.3		62	21.5	61	19.8		0	0	0	0	
*Age at randomisation (years)*					0.737					0.850					0.330
49–54	150	19.6	174	20.9		0	0	0	0		150	31.4	174	33.2	
55–59	229	29.9	238	28.6		67	23.3	66	21.4		162	33.9	172	32.8	
60–64	217	28.3	248	29.8		101	35.1	110	35.7		116	24.3	138	26.3	
65–69	133	17.4	128	15.4		85	29.5	88	28.6		48	10.0	40	7.6	
70–74	37	4.8	44	5.3		35	12.2	44	14.3		2	0.4	0	0	
*Smoking status*					2.95E − 08					2.49E − 10					
Never	69	9.0	153	18.4		69	24.0	153	49.7		0	0	0	0	
Former	167	21.8	128	15.4		167	58.0	128	41.6		0	0	0	0	
Current	530	69.2	551	66.2		52	18.1	27	8.8		478	100	524	100	
*Pack-years of Smoking*					8.41E − 13					3.43E − 11					2.37E − 08
< 1	70	9.1	154	18.5		70	24.3	154	50.0		0	0	0	0	
1–20	116	15.2	201	24.2		50	27.4	48	15.6		66	13.8	153	29.2	
21–40	270	35.2	235	28.2		56	29.4	51	16.6		214	44.8	184	35.1	
41–60	198	25.8	154	18.5		55	29.1	35	11.4		143	29.9	119	22.7	
> 60	109	14.2	85	10.2		54	18.8	17	5.5		55	11.5	68	13.0	
Missing	3	0.4	3	0.4		3	1.0	3	1.0		0	0	0	0	
Smoking duration (years)					3.17E − 09					7.12E − 10					3.28E − 04
0	69	9.0	153	18.4		69	24.0	153	49.7		0	0	0	0	
1–15	43	5.6	59	7.1		35	12.2	29	0.4		8	1.7	30	5.8	
16–30	150	19.6	172	20.7		62	21.5	57	18.5		88	18.4	115	21.7	
31–45	416	54.3	353	42.4		88	30.6	47	15.3		328	68.6	306	58.4	
46–60	78	10.2	52	6.2		31	10.8	17	5.5		47	10.2	35	6.6	
Missing	10	1.3	43	5.2		3	1.0	5	1.6		7	1.5	38	7.5	
Smoking intensity (packs/day)					6.48E − 07					1.27E − 10					0.0246
0	69	9.0	153	18.4		69	24.0	153	49.7		0	0	0	0	
> 0 and ≤ 1	415	54.2	399	48.0		116	40.3	90	29.2		299	62.6	309	59.0	
> 1 and ≤ 2	244	31.9	211	25.4		80	27.8	59	19.2		164	34.3	152	29.0	
> 2 and ≤ 3	30	3.9	27	3.2		22	7.6	3	1.0		8	1.7	24	4.6	
> 3 and ≤ 4	1	0.1	2	0.2		1	0.3	1	0.3		0	0	1	0.2	
Missing	7	0.9	40	4.8		0	0.0	2	0.6		7	1.5	38	7.3	

Results are presented for the full 1598 included participants, and for each of the contributing study separately

*p*-values were derived from a chi-square test investigating potential differences in the distribution of each factors in cases and controls

### Investigating the effect of smoking on bladder cancer

Of the 2670 CpG sites assayed in our data that were previously reported to be smoking-related, 200 CpG sites were found to be differentially methylated at a Bonferroni corrected significance level in our data. Principal component analysis of the methylation M-value at these CpG sites suggested that 71 components were necessary to explain more than 80% of the total variance, and the first component (PC1), alone, explained more than 19.3% of the variance (and the first 10 PC explain jointly 47% of the variance). Hypothesizing that PC1 provided a reasonable summary of the 200 smoking-related CpG, we used it as a proxy for the smoking-related CpG sites in subsequent smoking-adjusted analyses (the score was used as an adjustment variable). All smoking metrics were associated with risk of bladder cancer with OR ranging from 1.8 to 3.75 (Table 2, OR > 1.8 and *p*-value < $4.1\times {10}^{-14}$). After adjusting the model for the methylation level at cg05575921 (AHRR), the CpG site exhibiting the strongest association with smoking status in our data ($\beta$=− 1.943, *p*  < ${10}^{-100}$), results were attenuated with OR for the questionnaire-based smoking metrics ranging from 1.36 to 2.51, and corresponding *p*-values from $6.9\times {10}^{-2}$ to $1.4\times {10}^{-6}$ (Table 2). After adjusting our model for PC1, results were further attenuated (OR ranging from 1.32 to 2.47 and *p*-values from $9.1\times {10}^{-2}$ to $1.1\times {10}^{-6})$. Conversely, the risk of bladder cancer by quartiles of cg05575921 (AHRR) showed ORs ranging from 1.41 to 2.58 and *p*-values from $1.3\times {10}^{-2}$ to $5.7\times {10}^{-10}$ and for PC1 ORs ranging from 1.25 to 2.48, and corresponding *p*-values from $1.0\times {10}^{-1}$ to $1.6\times {10}^{-9}$. When adjusting for smoking metrics the ORs attenuated for both AHRR and PC1, which was most pronounced for smoking duration (2.58 to 1.47 and 2.48 to 1.63 when comparing Q4 versus Q1 for AHRR and PC1, respectively) (Table 3).

**Table 2:** Bladder cancer risk (Odd Ratio [OR] and 95% confidence intervals) by quartiles of smoking metrics

		Crude Model^a^		Adjusted on cg05575921 (AHRR)		Adjusted on PC1^b^	
		OR	*p*-value	OR	*p*-value	OR	*p*-value
Pack-years	Q1 vs Q2	1.823 (1.33, 2.50)	1.75e − 04	1.36 (0.98, 1.90)	6.95e − 02	1.328 (0.98, 1.89)	9.09e − 02
	Q1 vs Q3	2.981 (2.17, 4.11)	2.04e − 11	2.022 (1.43, 2.87)	7.38e − 05	2.013 (1.43, 2.87)	5.28e − 05
	Q1 vs Q4	2.933 (2.16, 4.00)	9.08e − 12	1.817 (1.28, 2.59)	9.24e − 04	1.751 (1.28, 2.56)	1.31e − 03
Duration (years)	Q1 vs Q2	2.283 (1.64, 3.20)	1.44e − 06	1.683 (1.18, 2.40)	3.97e − 03	1.628 (1.18, 2.40)	6.50e − 03
	Q1 vs Q3	3.751 (2.67, 5.30)	4.10e − 14	2.51 (1.73, 3.65)	1.36e − 06	2.474 (1.73, 3.65)	1.06e − 06
	Q1 vs Q4	3.465 (2.44, 4.95)	4.90e − 12	2.113 (1.42, 3.15)	2.36e − 04	2.061 (1.42, 3.15)	2.26e − 04
Intensity (packs/day)	Q1 vs Q2	2.703 (2.03, 3.62)	1.77e − 11	1.985 (1.46, 2.70)	1.15e − 05	2.036 (1.46, 2.70)	4.04e − 06
	Q1 vs Q3	2.636 (1.71, 4.07)	1.06e − 05	1.909 (1.22, 2.98)	4.43e − 03	1.913 (1.22, 2.98)	4.35e − 03
	Q1 vs Q4	2.551 (1.87, 3.48)	3.15e − 09	1.694 (1.21, 2.37)	2.10e − 03	1.657 (1.21, 2.37)	2.80e − 03

Results are also presented for the model additionally adjusted for the methylation level at cg05575921 (AHHR), the strongest CpG-site associated with smoking status in our dataset and on the first principal component (PC1) summarising the methylation levels at the 200 smoking-related CpG sites in our data

Quartile definition (sample size):

*Pack-years*: Q1: [0, 15[ (*n* = 383); Q2: [15, 31[ (*n* = 378); Q3: [31, 46[ (*n* = 381); Q4: [46, 184] (*n* = 403)

*Duration (years)*: Q1: [0, 20[ (*n* = 359); Q2: [20, 34[ (*n* = 391); Q3: [34, 40[ (*n* = 322); Q4: [40, 59] (*n* = 473)

*Intensity (packs/day)*: Q1: [0, 0.5[ (*n* = 300); Q2: [0.5, 1[ (*n* = 334); Q3: [1, 1.27[ (*n* = 522); Q4: [1.27, 4] (*n* = 389)

^a^All models were adjusted for age, sex and centre

^b^PC1 explains 19.3% of the total variance

**Table 3:** Bladder cancer risk (Odd Ratio [OR] and 95% confidence intervals) by quartiles of methylation methylation level at cg05575921 (AHHR) and the first principal component (PC1) summarising the methylation levels at the 200 smoking-related CpG site

		Crude Model^a^		Adjusted on pack-years		Adjusted on duration (years)		Adjusted on intensity (packs/day)	
		OR	*p*-value	OR	*p*-value	OR	*p*-value	OR	*p*-value
cg05575921 (AHRR)	Q2 vs Q1	1.41 (1.08, 1.85)	1.3e − 02	1.36 (1.04, 1.79)	2.6e − 02	1.36 (1.04, 1.79)	2.6e − 02	1.39 (1.06, 1.82)	1.8e − 02
	Q3 vs Q1	1.47 (1.12, 1.93)	5.4e − 03	1.29 (1.03, 1.7)	7.8e − 02	1.20 (1.12, 1.6)	2.3e − 01	1.39 (1.06, 1.84)	1.8e − 02
	Q4 vs Q1	2.58 (1.91, 3.48)	5.7e − 10	1.87 (1.34, 2.62)	2.8e − 04	1.47 (1, 2.19)	5.2e − 02	2.09 (1.52, 2.89)	7.5e − 06
PC1^b^	Q2 vs Q1	1.25 (1.04, 1.63)	1.0e − 01	1.18 (1.11, 1.54)	2.3e − 01	1.18 (1.11, 1.54)	2.2e − 01	1.21 (1.08, 1.57)	1.7e − 01
	Q3 vs Q1	1.89 (1.43, 2.5)	9.5e − 06	1.64 (1.23, 2.19)	8.7e − 04	1.58 (1.18, 2.12)	2.1e − 03	1.75 (1.31, 2.33)	1.3e − 04
	Q4 vs Q1	2.48 (1.85, 3.34)	1.6e − 09	1.90 (1.38, 2.63)	9.2e − 05	1.63 (1.15, 2.32)	6.3e − 03	2.07 (1.52, 2.84)	4.5e − 06

Results are also presented for the model additionally adjusted for smoking metrics

Number of observations per quartile:

*cg055792*: Q1 (*n* = 386); Q2: (*n* = 386); Q3 (*n* = 386); Q4: (*n* = 387)

*PC1*: Q1 (*n* = 386); Q2: (*n* = 386); Q3 (*n* = 386); Q4: (*n* = 387)

^a^All models were adjusted for age, sex and centre

^b^PC1 explains 19.3% of the total variance

### Investigating 2670 previously identified smoking-related CpGs and bladder cancer

**Fig. 1:**
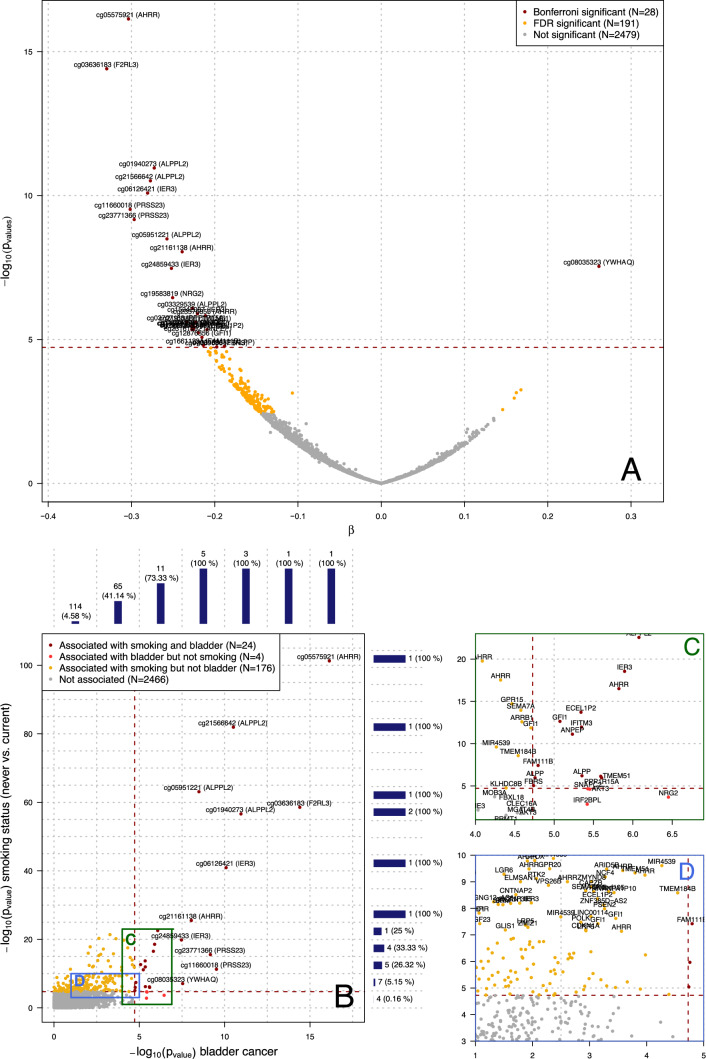
Results from the univariate analysis relating the methylation M-value at each of the 2,670 smoking-related CpG sites and the bladder cancer case/control status. The volcano plot (**a**) represents, for each of the 2670 CpG site separately, the effect size estimate (β; *X*-axis) representing the estimated methylation difference (on the logit scale) between cases and controls, and the *p*-value (*Y*-axis) for the null hypothesis of no association (${H}_{0}: \beta =0$) on the log_10_ scale. Horizontal red dashed line represents the Bonferroni-corrected significance level ensuring an FWER < 0.05 (*n* = 28). CpG sites found differentially methylated at an FDR level of 0.05 (*N* = 191) are presented in yellow. The associations between the 2670 smoking-related CpG sites and smoking status in our data are summarised in panel B by their *p*-values and are plotted against the *p*-value for the association with bladder cancer status. The (*n* = 200) CpG sites associated to smoking status are above the horizontal dashed line, which represent the Bonferroni-corrected significance level ensuring an FWER < 0.05. The (*N* = 24) CpG found associated to both smoking and bladder cancer status are presented in dark red, those exclusively associated to bladder cancer (*N* = 4) and smoking (*N* = 176) are plotted in light red and orange respectively. The marginal histogram along the axis summarise the number of CpG sites associated to bladder cancer (along the *Y*-axis) or to smoking (along the *X*-axis) in a given range of *p*-values for smoking (*Y* axis) and bladder cancer (*X*-axis). Panel C represents the 37 CpG sites with bladder *p*-values ranging from ${10}^{-10}$ and ${10}^{-4}$, and smoking *p*-values between ${10}^{-23}$ and ${10}^{-1}$. Among these, 17 are associated with both smoking and bladder cancer status, 4 are associated with bladder cancer but not smoking and 9 are associated with smoking but not bladder. Panel D represents the 92 CpG sites with bladder cancer *p*-values between ${10}^{-5}$ and ${10}^{-2}$ and smoking *p*-values between ${10}^{-17}$ and ${10}^{-4.5}$. Among these, 3 are associated with both smoking and bladder cancer and 82 are associated with smoking but not bladder cancer

Linear mixed models identified 28 differentially methylated smoking-related CpG sites in relation to bladder case–control status at a Bonferroni corrected significance level ($p=\frac{0.05}{\mathrm{2670}}=1.87 \times 10-05$) (Fig. 1a). Of these, 27 were hypo-methylated in prospective cases, and only cg08035323 (YWHAQ) was found hyper-methylated ($\beta$= 0.261 and *p*-value = 2.89e − 08). Stratifying the analyses by study (Supplementary Fig. 1a) we found that 8 of these 28 associations were significant in PLCO only, none in ATBC only, 3 in both PLCO and ATBC separately, and 17 were found statistically significant in the pooled analysis. The sign of the effect size estimates was highly consistent between the two studies (Supplementary Fig. 1b). Similarly, analyses restricted to current smokers from both PLCO and ATBC studies (*N* = 1100 participants) identified 5 differentially methylated sites at *p* < $1.87\times {10}^{-5}$, all of which were also identified in the full study population (Supplementary Fig. 2a). The strong consistency in the effect size estimates from the stratified analysis by study (Supplementary Fig. 2b) suggests that the signal attenuation we observe for smoking-associated CpGs between studies, may, at least partially, be attributed to less contrast in tobacco use due to the lack of non-smokers in ATBC.

Figure 1B represents smoking *p*-values (*Y*-axis) as a function of bladder cancer *p*-values (*Y*-axis) for the 2670 smoking-related CpG sites. The 6 most highly significant smoking CpGs were also the most highly associated with bladder cancer (smoking *p*-value < ${10}^{-40}$, bladder cancer *p*-value < ${10}^{-10}$). As indicated in Fig. 1b and c there were only 4 smoking-related CpG sites that were associated with bladder cancer but not smoking status in our data (*p*-ranging from $2.19\times {10}^{-5}$ to $1.47 \times {10}^{-3}$): cg11314684 (AKT3), cg19583819 (NRG2), cg13038618 (IRF2BPL), and cg14074174 (SNAPC2). We observed an additional 9 CpG sites with smoking *p*-values ranging from $1.7\times {10}^{-20}$ to $1.74\times {10}^{-5}$ that were borderline significantly associated with bladder case–control status (Fig. 1c* p*-value ranging from $1.97\times {10}^{-5}$ to $8.17\times {10}^{-5}$): cg18146737 (GFI1), cg10255761 (KLHDC8B), cg19859270 (GPR15), cg03991871 (AHRR), cg11902777 (AHRR), cg01901332 (ARRB1), cg01513913 (MIR4539), cg00310412 (SEMA7A), cg01127300 (TMEM184B). This represents a very small proportion (< 0.2%) of the CpG sites with smoking *p*-values > ${10}^{-5}$. That proportion dramatically increases for CpG sites with stronger associations with smoking. In particular, while 7/136 (5.15%) of the CpG sites with smoking *p*-values < 10^–10^ were associated with bladder cancer, 4/12 (33%) of the CpG sites in the $[{10}^{-15}, {10}^{-20}]$ smoking* p*-value bracket were associated with bladder cancer status, and all CpG sites with smoking* p*-values below ${10}^{-20}$ (*N* = 7) were associated with bladder cancer (Fig. 1b). Similarly, all CpG sites with bladder *p*-values below ${10}^{-7}$ (*N* = 11) were also associated with smoking status. Among the 36 CpG sites with bladder cancer *p*-values ranging from $3.16\times {10}^{-4}$ and ${10}^{-5}$, 22 were associated with smoking, and 4 were borderline significantly associated with smoking: cg04517079 (FOXP4), cg04263702 (FBXL18), cg15187398 (MOB3A), cg11436113 (SLC24A3) (Fig. 1d).

**Fig. 2:**
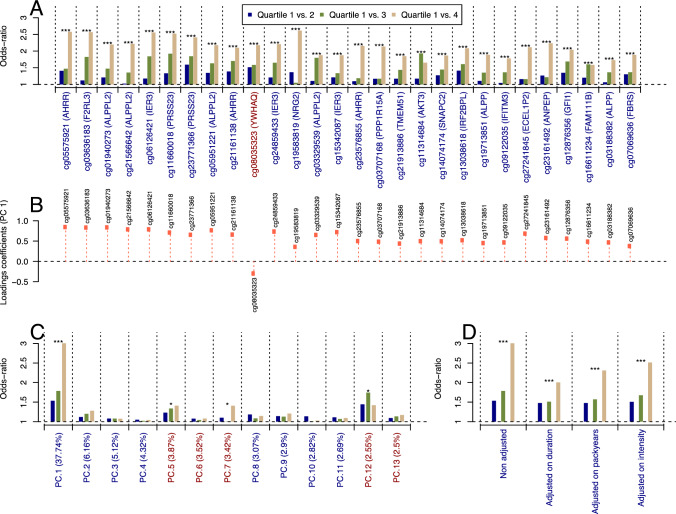
Odds ratios (ORs) calculated from the methylation M value at the 28 bladder-related CpG sites, which was recoded into quartiles. **a** The loadings coefficients of the first component of the Principal Component Analysis of the 28 methylation levels are presented in panel **b**. Using the same quartile discretisation for the scores of the 13 first components (jointly explaining 80.69% of the total variance), we calculated the OR for each component (panel **c**). The OR derived from the score of the first component were further adjusted for smoking duration, cumulative smoking exposure (in packyears), and smoking intensity (panel **d**). For all calculated OR, a linear model was used to test for a trend in the OR across methylation quartiles. For readability, corresponding *p*-values were coded as * for *p*-values in [0.05, 0.01], ** for *p*-values in [0.01, 0.001], and *** for *p*-values < 0.001. To ensure comparability across OR estimates, these were calculated setting the lowest quartile as reference, and derived the OR from the absolute value of the effect size estimate. As such, for CpG sites (or PC scores) found inversely associated to bladder cancer risk (marked in blue), the reported OR represents the risk change per-unit loss in methylation (or score), and for CpG sites found directly associated to disease risk (marked with a red), the OR represents the risk change per unit increase in methylation level (or score)

Methylation M-values of the 28 bladder-related CpG sites were recoded into quartiles, from which odds ratios were calculated (Supplementary Table 1). For each CpG site a clear risk gradient (*p*-trend < 0.001) across methylation quartiles was observed. ORs for the highest methylation quartile range from 1.58 to 2.63 for the 27 CpG sites found hypomethylated in cases, and OR = 2.17 for cg08035323 (YWHAQ) (Fig. 2a).

We conducted a principal component analysis (PCA) on the methylation M values at the 28 bladder-related CpG sites. Loading coefficients of the first component (explaining more than 37% of the original variance) were positive for all CpG sites except cg08035323 (YWHAQ) (Fig. 2b). We observed the same trend in ORs across the quartile of the scores of the first PC as with individual CpGs (Q4-Q1 OR = 3.01), and ORs by quartiles for the other PCs were weaker and did not exhibit any significant trend (except for PC5 and PC7) (Fig. 2c). ORs from the scores of the first component were further adjusted for smoking duration, cumulative smoking exposure (in packyears) and smoking intensity (Fig. 2d). All showed a similar pattern across quartiles and were slightly attenuated, in particular after adjusting for smoking duration. Analyses restricted to current smokers (*N* = 1100) showed similar results (Supplementary Fig. 3), but the attenuation upon adjustment for smoking duration was even less (Supplementary Fig. 3d, OR for the last quartile of PC1 scores adjusted on duration is 2.93 while it was 3.01 in the full population).

As a sensitivity analysis, we calculated the OR for each of the 28 bladder-related CpG sites (Supplementary Fig. 4), adjusting for smoking duration, cumulative smoking exposure, and smoking intensity. Results suggest that ORs are attenuated for all 28 CpG sites upon adjustment for smoking exposure, and that the attenuation is stronger while adjusting for smoking duration, irrespective of the CpG site.

### Epigenome-wide analyses of bladder cancer

**Fig. 3:**
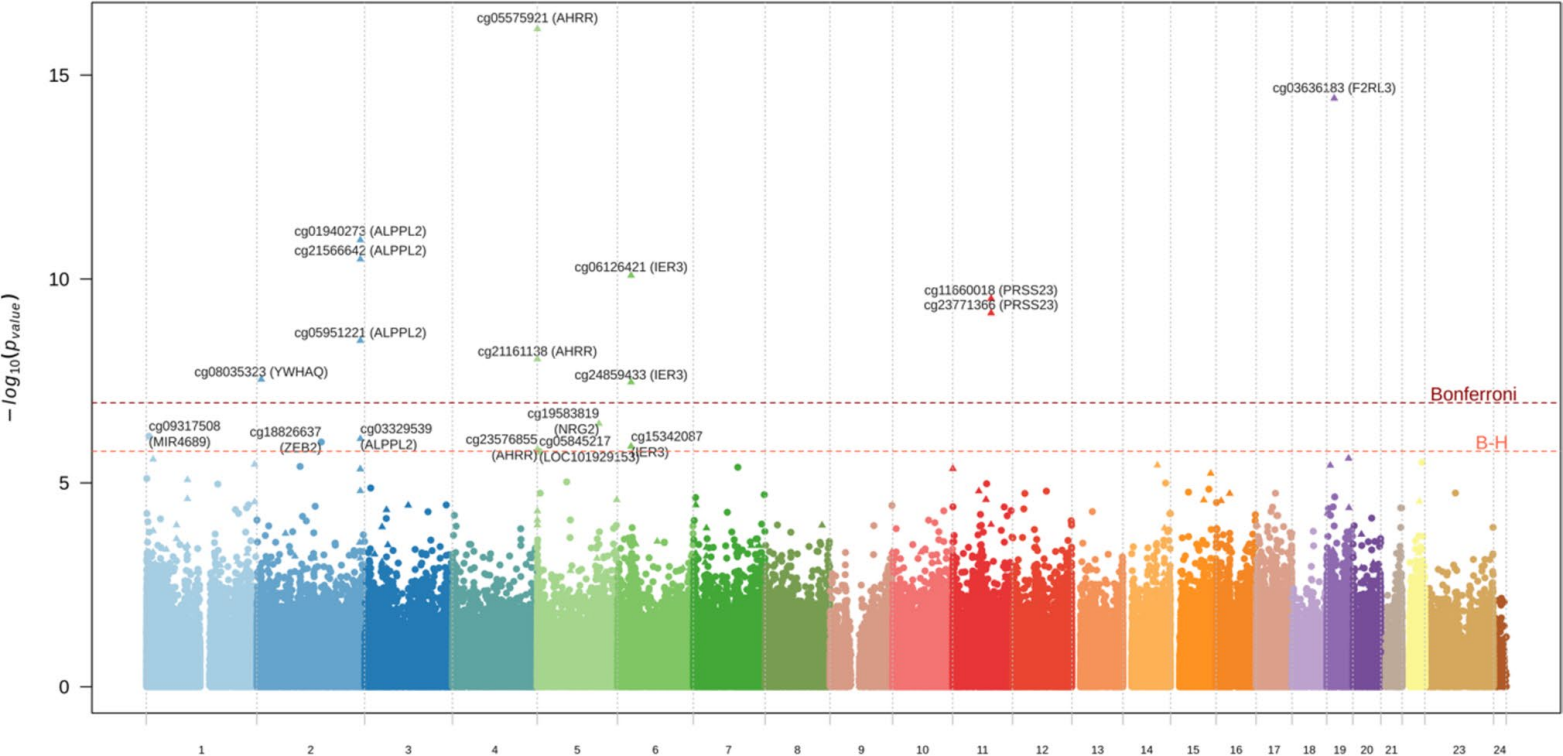
Manhattan plot summarising the full resolution association study relating the methylation M value at the 460,119 assayed CpG sites and bladder cancer case–control status. CpG sites that were found in the smoking-related analyses are represented by a triangle. Name and corresponding gene are only represented for the 11 differentially methylated CpG sites at a Bonferroni-corrected significance level and for the additional 7 differentially methylated sites with an FDR < 0.05

We compared bladder cancer cases with controls using the same univariate linear mixed model on the full set of CpGs and identified 11 differentially methylated CpG sites at a Bonferroni significance-corrected level ($p=\frac{0.05}{\mathrm{460,119}}=1.09 \times {10}^{-7}$), and 18 differentially methylated CpG sites while controlling the false discovery rate at 0.05 (Fig. 3). Of these 18 CpG sites, 15 were among those identified in our smoking-related analyses, while the remaining 3 cg09317508 (MIR4689), cg18826637 (ZEB2), and cg05845217 (LOC101929153) have not been systematically reported as being smoking-related in the literature. Epigenome-wide analyses restricted to current smokers (Supplementary Fig. 5) did not identify any differentially methylated CpGs (irrespective of the multiple testing correction used). However, the CpG sites identified in the full population were among the strongest associations in current smokers with consistent effect estimates compared to the full population with *p*-values ranging from ${1.82\times 10}^{-6}$ to $1.24\times {10}^{-2}$ (Supplementary Table 2). Further adjustement for blood cell composition did not affect our conclusions (result not shown).

### Investigating differentially methylated regions (DMRs)

**Fig. 4:**
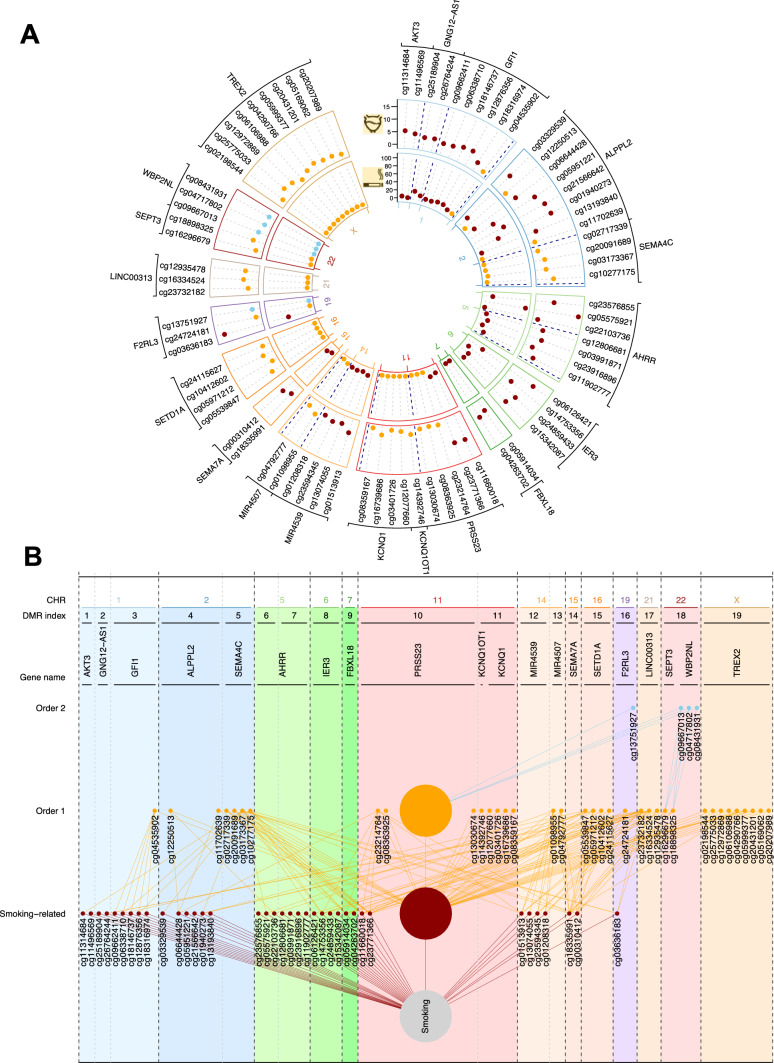
Description of the 19 identified Differentially Methylated Regions (DMR) in relation to Bladder cancer case–control status (**a**). For each of the 77 CpG sites included in the 19 DMRs, we report their *p*-value in relation to (i) smoking (inner circle), and bladder cancer (outer circle). CpG sites that are among the 2670 smoking-related CpG sites are coloured in dark red, CpG sites that are one order away from smoking are coloured in orange, and those two orders away from smoking, in blue. As depicted in panel **b**, of the 77 CpG sites included in the 19 identified DMRs, 37 are related to smoking, 36 one order away from smoking (i.e. correlated to at least one smoking-related CpG site but not smoking directly), and 4 correlated to at least one ‘order 1’ CpG site (second order). For clarity we represent all CpG sites that are not within the identified DMR and correlated to any CpG site in the identified DMRs as a single node in B (large nodes)

Differentially methylated regions analyses performed on the entire 450k CpG dataset identified 19 Differentially Methylated Regions containing 77 CpGs sites at an FDR level below 0.05 (Fig. 4a).

DMR included between 2 and 9 CpG sites each, with a length ranging from 18 to 2125 base pairs, and were located on chromosomes 1, 2, 5, 6, 7, 11, 14, 15, 16, 19, 21, 22 and X. Of these 19 DMRs, 5 included at least one of the 11 differentially methylated CpG sites identified in our univariate analyses; altogether 9 of the 11 genome-wide differentially-methylated CpG sites were located within the 19 DMRs.

The 19 DMRs included 77 CpG sites, of which 68 were not identified in our univariate analyses. Among these 77 CpG sites, 37 were among the 2670 smoking-related CpG sites, and the *p*-value for their association with smoking status in our data ranged from $5.17\times {10}^{-102}$ to $1.34\times {10}^{-1}$. For example, DMRs 4 and 6 contain cg21566642 near ALPPL2 and cg05575921 in AHRR, the two most highly significant CpGs for smoking. The remaining 40 CpG sites (located in DMRs 3–5, 10, 11,13, 15–19) were not directly related to smoking, and of these 35 ‘first order’ CpG sites were significantly correlated with at least one of the 2670 smoking-related CpG site, and 5 ‘second order’ CpG sites were correlated with at least one ‘first order’ CpG site but not directly with any smoking-related CpG site. Twelve DMRs include at least one smoking-related CpG site, and these may drive their association with bladder cancer. However, for the other 7 DMRs (i.e., numbers 5 (Chr2), 11 (Chr 11), 13 (Chr 14), 15 (Chr 16), 17 (Chr 21), 18 (Chr 22) and 19 (Chr X)), the distance to the smoking-bladder cancer-related CpGs within the DMR is equal to or more than 2 orders away from smoking, suggesting more distal, potentially non-tobacco associated processes related to bladder cancer (Fig. 4b).

### Prediction of bladder cancer

**Fig. 5:**
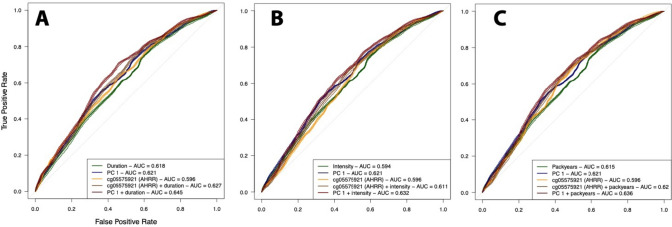
Receiver-Operating-Curve (ROC) analyses summarising the logistic model for smoking duration (**a**), smoking intensity (**b**), and pack-years (**c**), ROC curves are presented for the model including (i) the smoking metrics alone (green), (ii) the scores of the first principal component of the 28 CpG sites found differentially methylated in relation to smoking status (PC1 explaining 37.7% of the total variance, in blue), (iii) methylation levels at cg05575921 (AHRR), the CpG site exhibiting the strongest association with smoking status in our data (orange), (iv) methylation levels at cg05575921 and the smoking exposure metric (brown), and (v) PC1 scores and the smoking exposure measurement (dark red). We report the area under the curved from the testing set (20% of the total population) for each of the model investigated

ROC analyses (Fig. 5) showed that, irrespective of the smoking metric, cg05575921 (AHHR) alone (AUC 0.60) yielded similar predictive performances than the classical questionnaire-based smoking metrics (AUC 0.62. 0.59, 0.62 for duration, intensity, and packyears, respectively). A model including the PC1 from the 200 CpG sites differentially methylated in relation to smoking status in our study outperformed all other models (AUC 0.62). The best prediction was achieved by including PC1 and the smoking metrics in the model, resulting in an AUC slightly higher than those with PC1 only (Range AUC 0.63 to 0.65).

## Discussion

We conducted the largest prospective WBC DNA EWAS of bladder cancer, to the best of our knowledge, and found that CpG sites associated with risk of this tumor were predominantly related to smoking behaviour. We observed that the strongest smoking-associated DNA methylation CpGs were universally associated with future bladder cancer and that the majority of the identified differential methylation regions (DMRs) were directly or indirectly associated with smoking.

We identified 18 differentially methylated CpG sites of which 15 were among the smoking- related CpGs reported by Joehannnes et al. [9]. Of the three CpGs not included in our a-priori smoking-related CpGs, cg05845217 has been reported by Joehannes et al. to be differentially expressed with smoking status although with relatively lower statistical significance. cg18826637 has been reported by Yu et al. to be related to smoking status [6]. As such, only cg09317508 has not been previously reported to be associated with smoking. cg09317508 is positioned near the Nephrocytin 4 gene (NPHP4), a WNT pathway gene and is upstream of microRNA MIR4689, which has been reported to interact with KRAS and AKT in colorectal cancer [27].

We observed that adjusting the effect of the smoking-related CpGs on bladder cancer risk were only marginally attenuated when including questionnaire-based smoking metrics, similar to the report by Dugue et al. [28]. Similarly, we showed that the risk estimates of tobacco smoking duration, intensity, and packyears only marginally changed when including smoking-related CpGs, while prediction is improved. As such, smoking-related CpGs and clusters seem to capture other aspects of smoking behavior, exposure and biology that are not reflected by classical questionnaire-based metrics of smoking behavior that is relevant for bladder cancer risk. Notably, the largest attenuation in the effect of smoking-related CpGs on bladder cancer risk was seen when adjusting for duration of smoking with an attenuation effect that was about twice as strong as with intensity of smoking, consistent with the observation that tobacco smoking duration is the most important component of smoking behavior for bladder cancer risk [3].

Another interesting observation is that about 7% of the smoking-related CpGs were not associated with bladder cancer risk (*n* = 176 CpGs) in our study. Most of these CpGs had a weaker association with smoking in our dataset and as such the result can be partially explained by the lack of power to identify associations with bladder cancer. At the same time, several CpGs displayed relatively strong associations with smoking in our dataset but still were not associated with bladder cancer risk (i.e., cg06644428, cg23079012, cg19572487, cg14580211) suggesting that there might be some degree of specificity between CpG sites related to tobacco use and bladder carcinogenesis.

We searched for evidence of CpGs that are not related to smoking but did increase the risk of bladder cancer, although there was limited power to detect such associations given the small number of never-smokers in our study (Table 1) and the large contribution that smoking makes to bladder cancer etiology among smokers. We found that CpGs rs8102137 and rs224008 were marginally associated with bladder cancer risk but not with smoking, suggesting that CpG methylation might reflect other exogenous or endogenous exposures or biological processes relevant for bladder cancer etiology. Further, we identified 7 DMRs that did not contain any smoking-related CpG but included CpGs correlated in the 1st or 2nd order to smoking-related CpGs. DMR 18 on Chr 22 is linked to genes SEPT3 and WBP2NL. CpGs in both genes have not previously been linked to smoking behavior. Septine 3 (SEPT3) is highly expressed in brain tissue and plays a role in malignant brain tumors. Septine 3 forms together with septine 9 and 12 a sub-family of septins. Interestingly, SEPT9 has been proposed as a potential diagnostic target for the detection of urological cancers [29, 30]. WWP2 N-terminal-like (WBP2NL) is a testis-specific signaling protein that induces meiotic resumption and oocyte activation events and has been linked to breast cancer.

Our study has several limitations. We used a single biological sample that may not optimally represent the disease's exposure status and etiological time-window. In addition, although this is the largest study to date on the association between smoking, WBC DNA methylation, and bladder cancer the sample size is still relatively small for a study investigating a large number of hypotheses and we therefore cannot rule out that some of our findings are false-positives. We tried to protect against false-positive results by focusing our analyses on an established set of smoking-related markers, employing proper thresholds accounting for multiple comparisons, and testing consistency between the two studies. Our population comprised a relatively low number of never-smokers thus reducing exposure contrast in our study. Although this could have limited our analyses, it resulted in our reference category (1st quartile) not being solely non-smokers. The latter could have resulted in an increased probability of confounding as never and ever-smokers may differ in other factors that could not be taken into account in our analysis. Furthermore, as participants of both the PLCO Trial and the ATBC Study in this paper are Caucasian results may not be generalizable to populations with other demographic backgrounds.

In conclusion, our results confirm previous reports that blood-based DNA methylation markers related to future bladder cancer risk are largely driven by smoking behavior. However, these methylation markers also capture other aspects of smoking behavior than are typically assessed in questionnaires. We also identified some putative markers that are not related to smoking but yet appear to be associated with future bladder cancer risk. These findings, which require replication, can further our understanding of the etiology of bladder cancer and potentially contribute to future risk prediction models that incorporate other environmental as well as genetic risk factors.

### Supplementary Information

Below is the link to the electronic supplementary material.

Supplementary file1 (DOCX 2371 kb)
