# Supplementary material for: A prospective study of smoking-related white blood cell DNA methylation markers and risk of bladder cancer
Source: Eur J Epidemiol. 2024 Mar 30;39(4):393–407. doi: 10.1007/s10654-024-01110-y (PMC11101379; doi:10.1007/s10654-024-01110-y)
Supplement: Supplementary file 1 — Supplementary file1 (DOCX 2371 kb) [file 10654_2024_1110_MOESM1_ESM.docx]

**Supplementary Tables**

|  | Q2 | Q3 | Q4 |
| --- | --- | --- | --- |
| cg05575921 (AHRR) | 1.41 | 1.471 | 2.577 |
| cg03636183 (F2RL3) | 1.117 | 1.825 | 2.569 |
| cg01940273 (ALPPL2) | 1.207 | 1.472 | 2.196 |
| cg21566642 (ALPPL2) | 1.022 | 1.357 | 2.215 |
| cg06126421 (IER3) | 1.177 | 1.841 | 2.553 |
| cg11660018 (PRSS23) | 1.333 | 1.922 | 2.523 |
| cg23771366 (PRSS23) | 1.593 | 1.845 | 2.409 |
| cg05951221 (ALPPL2) | 1.345 | 1.632 | 2.176 |
| cg21161138 (AHRR) | 1.388 | 1.702 | 2.098 |
| cg08035323 (YWHAQ)* | 1.516 | 1.583 | 2.178 |
| cg24859433 (IER3) | 1.207 | 1.65 | 2.209 |
| cg19583819 (NRG2) | 1.365 | 1.05 | 2.614 |
| cg03329539 (ALPPL2) | 1.104 | 1.798 | 1.879 |
| cg15342087 (IER3) | 1.209 | 1.336 | 1.874 |
| cg23576855 (AHRR) | 1.095 | 1.186 | 2.154 |
| cg03707168 (PPP1R15A) | 1.166 | 1.172 | 2.133 |
| cg21913886 (TMEM51) | 1.172 | 1.434 | 1.847 |
| cg11314684 (AKT3) | 1.175 | 1.929 | 1.652 |
| cg14074174 (SNAPC2) | 1.274 | 1.444 | 1.864 |
| cg13038618 (IRF2BPL) | 1.414 | 1.609 | 2.082 |
| cg19713851 (ALPP) | 1.106 | 1.351 | 1.887 |
| cg09122035 (IFITM3) | 1.044 | 1.364 | 1.783 |
| cg27241845 (ECEL1P2) | 1.157 | 1.157 | 2.115 |
| cg23161492 (ANPEP) | 1.267 | 1.217 | 2.234 |
| cg12876356 (GFI1) | 1.347 | 1.688 | 2.035 |
| cg16611234 (FAM111B) | 1.201 | 1.604 | 1.583 |
| cg03188382 (ALPP) | 1.063 | 1.364 | 1.73 |
| cg07069636 (FBRS) | 1.302 | 1.367 | 1.884 |

**Supplementary Table 1** Odds ratios (ORs) for the methylation level (recoded in quartile) at each of the 28 CpG sites found differentially methylated in cases and controls at a Bonferroni corrected significance level. To ensure comparability of estimates across CpG sites, OR were calculating setting the lowest quartile as reference, and derived the OR from the absolute value of the effect size estimate. As such, for CpG sites found hypomethylated in cases, the reported OR represents the risk change per-unit loss in methylation, and for CpG sites found hypermethylated in cases (marked with a star), the OR represents the risk change per unit increase in methylation level.

|  | Full sample | | | Current smokers | | |
| --- | --- | --- | --- | --- | --- | --- |
|  | N | β | p-value | N | β | p-value |
| cg05575921 (AHRR) | 1598 | -0.3 | 7.3e-17 | 1081 | -0.17 | 4.8e-06 |
| cg03636183 (F2RL3) | 1597 | -0.33 | 4.0e-15 | 1081 | -0.2 | 2.4e-05 |
| cg01940273 (ALPPL2) | 1598 | -0.27 | 1.1e-11 | 1081 | -0.14 | 4.3e-04 |
| cg21566642 (ALPPL2) | 1590 | -0.28 | 3.1e-11 | 1081 | -0.18 | 9.9e-06 |
| cg06126421 (IER3) | 1598 | -0.28 | 8.2e-11 | 1081 | -0.23 | 1.8e-06 |
| cg11660018 (PRSS23) | 1598 | -0.3 | 3.0e-10 | 1081 | -0.25 | 1.1e-05 |
| cg23771366 (PRSS23) | 1598 | -0.3 | 6.8e-10 | 1081 | -0.23 | 2.7e-05 |
| cg05951221 (ALPPL2) | 1597 | -0.26 | 3.2e-09 | 1081 | -0.15 | 2.8e-04 |
| cg21161138 (AHRR) | 1597 | -0.24 | 9.0e-09 | 1081 | -0.15 | 6.9e-04 |
| cg08035323 (YWHAQ) | 1598 | 0.26 | 2.9e-08 | 1081 | 0.24 | 2.1e-05 |
| cg24859433 (IER3) | 1598 | -0.25 | 3.4e-08 | 1081 | -0.16 | 5.2e-03 |
| cg19583819 (NRG2) | 1598 | -0.25 | 3.5e-07 | 1081 | -0.17 | 6.4e-04 |
| cg09317508 (MIR4689) | 1595 | -0.24 | 6.9e-07 | 1081 | -0.17 | 2.4e-04 |
| cg03329539 (ALPPL2) | 1597 | -0.23 | 8.4e-07 | 1081 | -0.13 | 2.0e-03 |
| cg18826637 (ZEB2) | 1594 | -0.24 | 9.3e-07 | 1081 | -0.24 | 4.9e-05 |
| cg15342087 (IER3) | 1598 | -0.22 | 1.3e-06 | 1081 | -0.16 | 4.1e-03 |
| cg23576855 (AHRR) | 1598 | -0.21 | 1.5e-06 | 1081 | -0.16 | 9.8e-04 |
| cg05845217 (LOC101929153) | 1596 | -0.24 | 1.6e-06 | 1081 | -0.15 | 1.2e-02 |

**Supplementary Table 2** Results from the linear models regressing the methylation level (set as the outcome variable) as a function of bladder cancer case control status. Results are presented for the model fitted on the full population and on current smokers only. Results are presented for the 18 CpG sites found differentially methylated in cases and controls at an FDR level < 0.05.

**Supplementary figures**

**Supplementary Figure 1** Comparison of the p-values (panel A) and effect size estimates (panel B) from the linear mixed model regressing the methylation M values against the bladder cancer case control status for the 2,670 smoking-related CpG sites obtained in PLCO and ATBC studies separately. Results are presented for the ATBC (X-axis) and PLCO studies (Y-axis). Diagonal line is the X=Y-line. For clarity only the 28 CpG sites found differentially methylated in the full population are named in the plots.

**Supplementary Figure 2** Comparison of the p-values (panel A) and effect size estimates (panel B) from the linear mixed model regressing the methylation M values against the bladder cancer case control status for the 2,670 smoking-related CpG sites obtained in the full population (X-axis) and in current smokers only (Y-axis). Diagonal line is the X=Y-line. For clarity only the 28 CpG sites found differentially methylated in the full population are named in the plots.

**Supplementary Figure 3** Odds ratios (ORs) calculated from the methylation M value at the 28 bladder-related CpG sites, which was recoded into quartiles (A). Results are presented restricting the study population to (N=1,036) current smokers. The loadings coefficients of the first component of the Principal Component Analysis of the 28 methylation levels are presented in panel B. Using the same quartile discretisation for the scores of the 13 first components (jointly explaining 80.69% of the total variance), we calculated the OR for each component (panel C). The OR derived from the score of the first component were further adjusted for smoking duration, cumulative smoking exposure (in packyears), and smoking intensity (panel D). For all calculated OR, a linear model was used to test for a trend in the OR across methylation quartiles. For readability, corresponding p-values were coded as * for p-values in [0.05, 0.01], ** for p-values in [0.01, 0.001], and *** for p-values < 0.001. To ensure comparability across OR estimates, these were calculated setting the lowest quartile as reference, and derived the OR from the absolute value of the effect size estimate. As such, for CpG sites (or PC scores) found inversely associated to bladder cancer risk (marked in blue), the reported OR represents the risk change per-unit loss in methylation (or score), and for CpG sites found directly associated to disease risk (marked with a red), the OR represents the risk change per unit increase in methylation level (or score).

**Supplementary Figure 4** Odds ratios (ORs)calculated from the methylation M value at the 28 bladder-related CpG sites, which was recoded into quartiles. To ensure comparability across OR estimates, these were calculated setting the lowest quartile as reference, and derived the OR from the absolute value of the effect size estimate. As such, for CpG sites found inversely associated to bladder cancer risk (marked in blue), the reported OR represents the risk change per-unit loss in methylation, and for CpG sites found directly associated to disease risk (marked with a red), the OR represents the risk change per unit increase in methylation level. Results are presented for models unadjusted (A), adjusted for smoking duration (B), cumulative smoking exposure (C), and smoking intensity (D). For all calculated ORs, a linear model was used to test for a trend in the OR across methylation quartiles. For readability, corresponding p-values were coded as * for p-values in [0.05, 0.01], ** for p-values in [0.01, 0.001], and *** for p-values < 0.001.

**
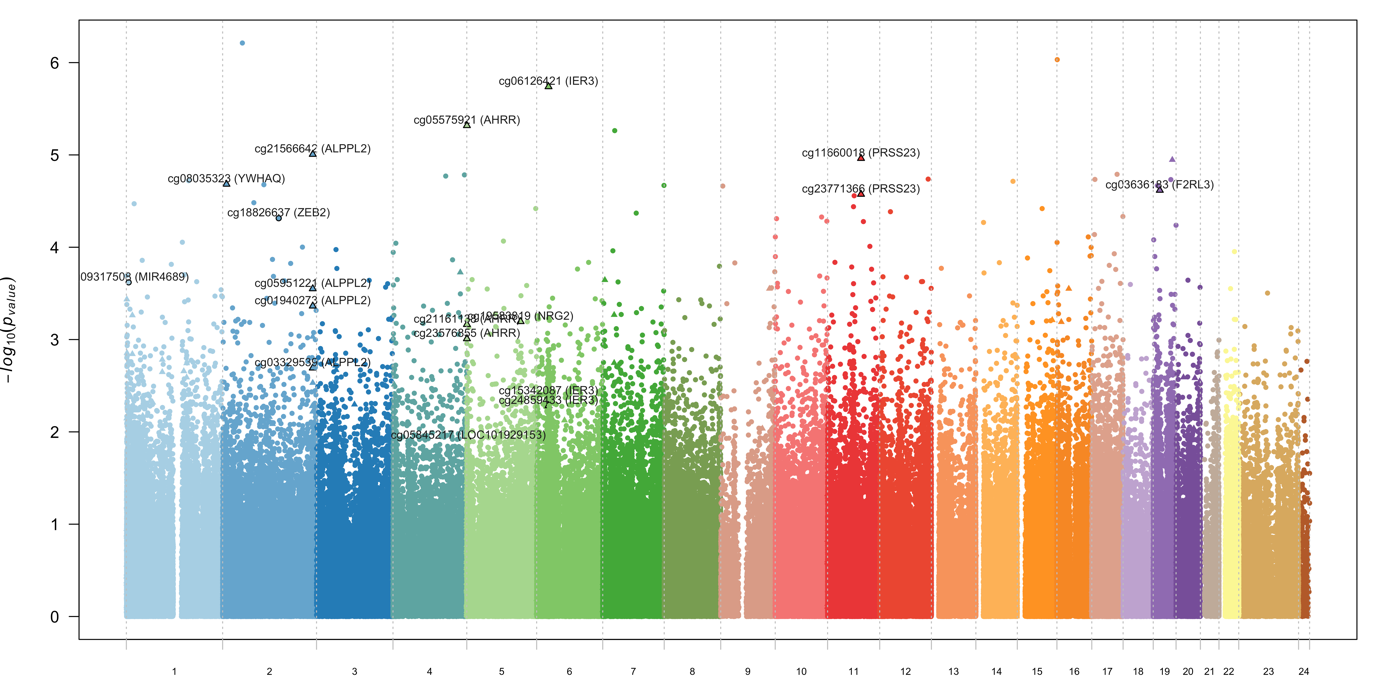
Supplementary Figure 5:** Manhattan plot summarising the full resolution association study relating the methylation M value at the 460,119 assayed CpG sites and bladder cancer case-control status. Results are presented for the analyses restricted to current smokers. CpG sites that were found in the smoking-related analyses are represented by a triangle. Name and corresponding gene are only represented for the 18 differentially methylated CpG sites at a controlled FDR level of 0.05.
